# Structural Insights into 4,5-DOPA Extradiol Dioxygenase from *Beta vulgaris*: Unraveling the Key Step in Versatile Betalain Biosynthesis

**DOI:** 10.1021/acs.jafc.4c09501

**Published:** 2025-03-07

**Authors:** Chih-Chia Chiang, Yen-Ju Lu, Jia-Wei Liu, Sheng-Wei Lin, Chun-Chi Chou, Chia-Hsin Lin, I-Weh Chien, Chun-Hua Hsu

**Affiliations:** a Department of Agricultural Chemistry, National Taiwan University, Taipei 10617, Taiwan; b Institute of Biochemical Sciences, National Taiwan University, Taipei 10617, Taiwan; c Genome and Systems Biology Degree Program, National Taiwan University and Academia Sinica, Taipei 10617, Taiwan; d Center for Computational and Systems Biology, National Taiwan University, Taipei 10617, Taiwan

**Keywords:** betalains, betalamic acid, extradiol dioxygenase, crystal structure

## Abstract

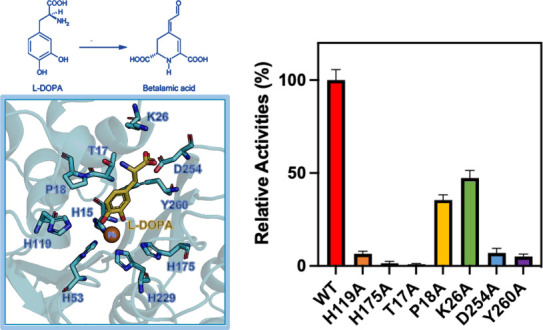

Betalains, a group of pigments widely distributed in various plants, are extensively applied in the food, beverage, and medicinal industries. The biosynthesis of betalains involves the enzymatic action of 4,5-DOPA-dioxygenase, which catalyzes the key ring-opening reaction of DOPA to produce betalamic acid, a crucial intermediate in the pathway. The crystal structure of a 4,5-DOPA-dioxygenase from *Beta vulgaris* (BvDOD) was determined in this study. The structural analysis revealed that BvDOD exhibited a structural fold similar to that of other members of the extradiol dioxygenase family. Moreover, the Fe-ligand residues His15, His53, and His229 indicated the enzyme’s reliance on nonheme iron for catalyzing the ring-opening reaction. Molecular docking and mutational analysis identified two conserved residues, His119 and His175, in the active site essential for the catalytic reaction. In addition, Thr17, Asp254, and Tyr260 contributed to properly positioning the substrate in the active site. This study has provided structural insights into substrate recognition and catalytic mechanisms of BvDOD, which can be applied to develop enzymes for improved betalain production.

## Introduction

Beetroot (*Beta vulgaris* L.) is a traditional vegetable that is widely distributed globally and is renowned for its commercial use in producing red juice and natural pigment.^1^ Beetroot is recognized as a highly nutritious vegetable and ranks among the top 10 vegetables in terms of antioxidant activity.^2^ The vibrant color of beetroot is attributed to betalains, water-soluble pigments stored in its cell vacuoles.^3^ Betalains are responsible for certain fruits, vegetables, and flowers’ vivid red–violet to yellow hues. These pigments have garnered attention owing to their potential as natural colorants and beneficial effects on human health. Furthermore, betalains play a protective role in plants and aid them in withstanding biotic and abiotic stresses.^4−6^ Occurring predominantly in plants of the Caryophyllales order, betalains are classified into red–purple betacyanins and yellow betaxanthins.^7−9^ Both share a common precursor molecule, betalamic acid, which acts as a chromophore. When betalamic acid is conjugated with amines, it forms betaxanthin; in contrast, conjugation with cyclo-DOPA glucoside forms betacyanin. This chromophore’s resonance system is critical for the biological activity of betalains.^10−12^ Nonetheless, compared with other plant pigments such as chlorophyll, carotenoids, and anthocyanins,^3,13^ the biosynthetic pathway of betalains has only been partially understood.

DOPA-4,5-dioxygenase (DOD) is an enzyme that catalyzes extradiol aromatic ring cleavage.^14,15^ These ring-cleaving dioxygenases play crucial roles in the aerobic microbial degradation of aromatic compounds.^11,16,17^ Various pathways converge in catecholic intermediates, which are subjected to ortho or meta cleavage by intradiol or extradiol dioxygenases, respectively. DOD has been widely applied in biotechnology, including microbial sensors for detecting copper ions^18−21^ and the synthesis of diverse betaxanthins by catalyzing the production of betalamic acid, followed by a Schiff condensation reaction with amines or amino acids.^22,23^

Although 4,5-DOPA-extradiol-dioxygenases have been studied for decades, their three-dimensional structure has not been elucidated. The DOD from *B. vulgaris* (BvDOD), the best-known betalain-producing plant, is indispensable in synthesizing sugar beet pigments.^24−26^ This potential coloring enzyme can be applied in detective reagents and in producing natural pigments. In this study, the properties of BvDOD were characterized, and the first crystal structure of this plant-type DOPA-dioxygenase was determined. In combination with modeling and mutational analysis, several key amino acids were identified in the catalytic process. Biochemical and structural analysis indicated that BvDOD shares a similar catalytic mechanism with extradiol dioxygenase members.

## Materials and Methods

### Materials

*Escherichia coli* strain BL21 (DE3) and the pET-21a plasmid (Novagen, Madison, WI, USA) were used for protein expression. Restriction endonucleases and T4 DNA ligase were purchased from Thermo Fisher Scientific (Hudson, NH, USA). PrimeSTAR HS DNA polymerase was purchased from Takara Bio (Otsu, Japan). All other chemicals and reagents were purchased from Sigma-Aldrich Chemical Co. (St Louis, MO, USA).

### Protein Expression and Purification

The DNA sequence encoding BvDOD (GenBank accession number: AJ583017) was synthesized (MDbio, Taiwan) and inserted into the pUC57 plasmid. Subsequently, the gene fragment of BvDOD was inserted between the NdeI and XhoI sites of the pET-21a vector system (Novagen). BvDOD mutants were constructed using site-directed mutagenesis (Agilent, Santa Clara, CA, USA). The primers used for the mutagenesis and mutant confirmation are listed in Table S1. Site-directed mutagenesis was performed using 14 polymerase chain reaction cycles. The resulting amplicon mixtures were digested using DpnI and transformed into *E. coli* cells via heat shock. The transformants were selected on Luria–Bertani medium containing 100 μg/mL ampicillin following overnight incubation at 37 °C.

The resulting plasmid with the inserted sequence was then transformed into *E. coli* BL21 (DE3). The cells were grown at 37 °C with 50 μg/mL of ampicillin until an OD 600 of 1.0 was reached. The expression of the recombinant BvDOD with a His-tag at the N terminus was induced in the cells using 1 mM isopropyl-β-D-thiogalactoside, followed by growth for 20 h at 25 °C. The cells were collected via centrifugation and resuspended in lysis buffer (25 mM Tris-HCl buffer, pH 7.0, 100 mM NaCl). After 20 min of sonication, the cell extract was clarified via centrifugation at 12,000 rpm for 20 min at 4 °C to remove the debris. The clear supernatant was loaded onto an open column packed with Ni-NTA resin. The resin was washed sequentially with lysis buffer containing 50 mM and 100 mM imidazole. His-tagged BvDOD was eluted using lysis buffer with 200 mM imidazole. The desired fractions were identified using sodium dodecyl sulfate-polyacrylamide gel electrophoresis, pooled, concentrated to <0.5 mL, and subjected to gel filtration chromatography to obtain a homogeneous protein and determine the molecular mass of BvDOD in solution.

### Gel Filtration Chromatography

Gel filtration chromatography (Superdex75 XK 16/60 column, GE Healthcare) using an AKTA FPLC system was used for further purification and examination of the oligomeric state of BvDOD. Chromatography was performed using 20 mM Tris-HCl buffer (pH 7.0) and 100 mM NaCl as the mobile phase at a flow rate of 0.1 mL/min. The calibration curve was constructed using gel filtration molecular weight standards containing gamma-globulin (158.0 kDa), ovalbumin (44.0 kDa), myoglobin (17.0 kDa), and vitamin B12 (1.35 kDa). The curve was linear in the 1.35–158 kDa range.

### Size-Exclusion Chromatography with Multiangle Light Scattering (SEC-MALS) Analysis

SEC-MALS analysis of BvDOD protein was performed to determine molecular weight and quaternary structure. The column (Enrich Tm SEC. 70 10 × 300, Bio-Rad Laboratories, Santa Barbara, CA, USA) was used with a flow rate of 0.5 mL/min in the buffer system of 20 mM Bis-Tris and 50 mM NaCl at pH 6.0 and 25 °C. Ultraviolet–visible (UV) (QELS, Wyatt Technology, Santa Barbara, CA, USA), static light scattering (mini DAWN TREOS, Wyatt Technology, Santa Barbara, CA, USA), quasi-elastic light scattering (QELS, Wyatt Technology, Santa Barbara, CA, USA), and refractive index (Optilab T-rEX, Wyatt Technology, Santa Barbara, CA, USA) detectors were aligned with the column. Bovine serum albumin (Sigma, A1900, Saint Louis, MO, USA) was used as a standard for calibration and optimization. The molecular weight was calculated using ASTRA 6 with the d*n*/d*c* value set to 0.185 mL g^–1^.

### Circular Dichroism (CD) Spectroscopy

CD measurement was conducted in a Chirascan-plus qCD spectrometer (Applied Photophysics, UK). Protein samples were prepared in 20 mM sodium phosphate at various pH values (5.5–9.0). The far-UV spectrum was acquired at 25 °C with a 20 μM protein sample in a 1 mm path-length cuvette. The 190–260 nm signals were recorded three times with a scan rate of 20 nm/min and a bandwidth of 1 nm after subtracting the blank signals from the solvent.

### High-Performance Liquid Chromatography (HPLC) and Mass Spectroscopy (MS) Analysis

A Hitachi HPLC system with a UV–vis detector was used for analytical HPLC separations. Reversed-phase chromatography was performed with the Purospher STAR RP-18 end-capped (4.6 × 150 mm, 5 μm) column (Merck, Germany). The mobile phase was 25 mM phosphate buffer of pH 6.0. The flow rate was 1 mL/min and was operated at 20 °C. The injection volume was 20 μL. Betalamic acid structure was confirmed via electrospray ionization mass spectrometry in an Agilent VL 1100 apparatus with an LC/MSD Trap (Agilent Technologies, Palo Alto, CA, USA).

### Enzyme Activity Assay

An in vitro assay was performed to ascertain the optimal temperature and pH for BvDOD activity. Enzyme activity was determined by measuring the production of betalamic acid at λ = 424 nm^27−29^ using the SpectraMax iD5Microplate Reader. The purified BvDOD was prepared in a reaction solution containing 25 mM sodium phosphate buffer, 2.5 mM L-DOPA, 0.5 mM FeCl_2_, and 10 mM ascorbic acid. To establish the optimal pH value, sodium phosphate (25 mM) was used for pH values ranging from 4.0 to 9.0. The dry bath machine was used to control the reaction temperature. BvDOD displayed the highest activity at 50 °C and pH 8.5.

To evaluate the effect of metal ions on BvDOD, the purified enzyme was preincubated for 1 h with the following salts: 1 mM each of CaCl_2_, CoCl_2_, CuSO_4_, FeSO_4_, Fe_2_(SO_4_)_3_, MgSO_4_, MnCl_2_, and NiSO_4_. Enzyme kinetics were determined in the reaction solution using 25 mM sodium phosphate buffer pH 8.5, 0.5 mM FeCl_2_, and 10 mM ascorbic acid. Substrate concentration was prepared from 2.5 mM to 0.039 mM and incubated with 20 μM BvDOD. The absorbance of λ = 424 nm was measured every 30 s using the SpectraMax iD5Microplate Reader (Molecular Devices, San Jose, CA, USA). The kinetics result was analyzed and fitted to the Michaelis–Menten equation. Each activity assay was performed in triplicate for validation.

### Crystallization and Data Collection

Initial protein crystallization trials were performed at 283 K using the sitting-drop vapor-diffusion method with commercial crystallization screen kits, 96-well Intelli-plates, and a Crystal Phoenix robot (Art Robbins Instruments). Each crystallization drop was prepared by mixing 0.3 μL BvDOD at 10 mg/mL with an equal volume of mother liquor. The mixture was equilibrated against a 100 μL reservoir solution. Crystals for data collection were grown in a solution containing 0.2 M ammonium phosphate monobasic, 0.1 M Tris (pH 8.5), and 50% (w/v) MPD over 1 week at 283 K. Following crystallization, Fe K-edge X-ray absorption spectroscopy scans were performed at the TPS05A beamline (National Synchrotron Radiation Research Center NSRRC, Taiwan) to determine the metal content and coordination environment within the crystals.

The crystal was cryoprotected in mother liquor supplemented with 20% glycerol and flash-frozen in liquid nitrogen at 100 K for subsequent diffraction. The X-ray diffraction images were acquired in a 100-K nitrogen gas stream using the ADSC Quantum-315r CCD Area Detector on the BL13B1 beamline (NSRRC, Taiwan). Individual frames comprised a 1° oscillation angle measured for 5 s at a crystal-to-detector distance of 250 mm. The crystal belonged to trigonal *P*3_1_21, with the following unit cell dimensions: a = 95.873, b = 95.873, and c = 124.908. Intensity data were processed with the HKL2000 software.^30^

### Structure Determination and Refinement

The crystal structure of BvDOD was elucidated using the molecular replacement method with the Phaser^31^ of the PHENIX suite of programs.^32^ The ygiD protein structure protein data bank (PDB) code: 2PW6; sequence identity: 37%) was used as the search model. The initial model was rebuilt interactively by inspecting the σ-weighted electron density maps with coefficients 2*mF*_o_-*DF*_*c*_ and *mF*_*o*_*-DF*_*c*_ in COOT^33^ and subsequently refined with phenix.refine^34^ until R_work_ and R_free_ values converged. The geometric parameters of the final models were verified using the PROCHECK^35^ and MolProbity programs.^36^ The volume of active sites was calculated using CAVER.^37^ Molecular visualizations were generated with PyMOL (The PyMOL Molecular Graphics System, version 1.7, Schrödinger, LLC).

### Iron Analysis

The enzyme was digested in 6N HNO_3_, and the iron content was quantified using inductively coupled plasma-mass spectrometry (ICP-MS) (Agilent 7700e, USA). The glassware used in the procedure was thoroughly acid-washed to prevent contamination.

### Small-Angle X-ray Scattering (SAXS)

SAXS was performed to explore the shape and quaternary structure of BvDOD. SAXS measurements were conducted at the TPS-13A BioSAXS beamline (NSRRC, Taiwan). Purified BvDOD was prepared in Tris buffer (20 mM Tris-HCl pH8, 100 mM NaCl) and exposed to X-rays with an online HPLC system. The concentration of the sample was 16.7 mg/mL. A total of 119 SAXS profiles were then collected with a detecting system comprising two detectors, namely, Eiger X 9 M and Eiger X 1 M. Protein samples were placed in a quartz capillary with a diameter of 2.0 mm using an exposure time of 1 s/frame. With an X-ray beam energy of 15.0 keV and a sample-to-detector distance of 2300.26 mm, the data covered a wide scattering vector q-range of 0.006–1.8 Å^–1^ (q is the momentum transfer vector and is defined as θ π λ q sin 4, where 2θ is the scattering angle and λ is the wavelength of X-rays). The experimental data (buffer subtraction, averaging, merging, gyration radius calculation, Guinier analyses, p(r) construction, bead modeling, and molecular model validation) were analyzed using the ATSAS program.^38^ The experimental SAXS data of BvDOD were fitted to the crystal structure using the CRYSOL program.^39^ The envelope model was constructed using the DAMMIF program^40^ and the WAXSiS web server.^41^ The envelope diagram-fitted crystal structure was produced using the SASpy tool,^42^ a PyMOL software plugin tool.

### Molecular Docking

Molecular docking of L-DOPA into the active site of BvDOD was conducted with the AutoDock software.^43^ The docking was performed in a box with an edge length of 15 Å around iron, which included the active site and its surroundings. The BvDOD structure with L-DOPA, which the diol positioned to iron, was applied to the YASARA energy minimization server.^44^

## Results

### Biochemical and Biophysical Characterization of BvDOD

**Figure 1:**
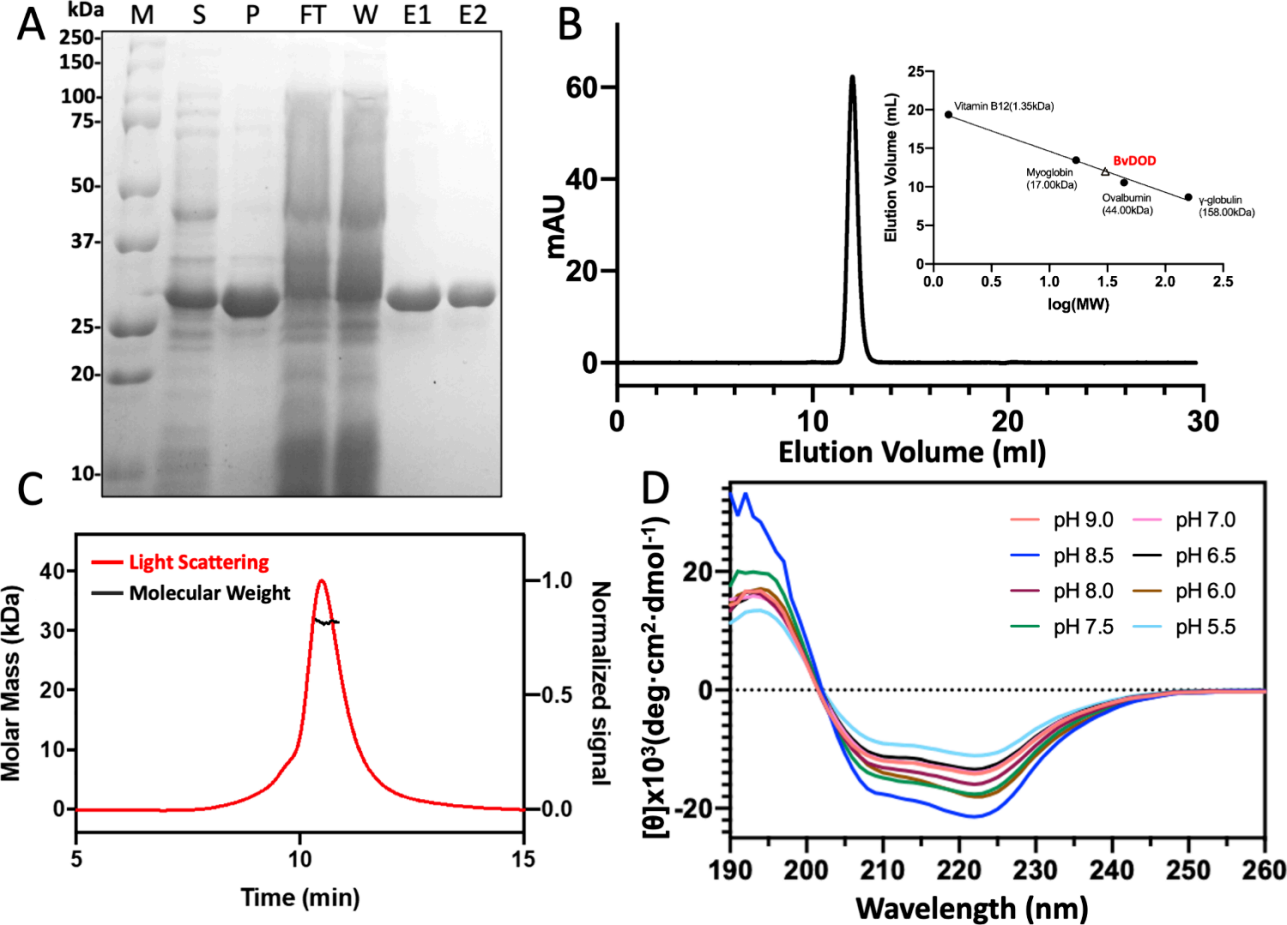
**Molecular properties of BvDOD** (A) SDS-PAGE analysis showing the expression and purification of recombinant BvDOD protein in bacteria. M, marker; S, supernatant; P, pellet; FT, flow-through; W, wash with 20 mM imidazole; E1, elution with 100 mM imidazole; E2, elution with 150 mM imidazole. (B) Analytical gel filtration chromatography of BvDOD. The calibration curve used to estimate the native molecular weight based on the elution position is indicated. (C) Size-exclusion chromatography coupled with multiangle static light scattering (SEC-MALS) analysis showing that BvDOD exists as a monomer in solution with a molecular weight of 30.5 kDa. (D) Far-UV circular dichroism (CD) spectra (195–260 nm) of BvDOD at various pH values.

BvDOD was cloned into the pET-21a vector to obtain recombinant proteins possessing N-terminal 6xHis tags. The proteins were well expressed in a soluble form in *E. coli* BL21 (DE3) cells. Ni-NTA column was used for affinity purification. As expected, the protein band for BvDOD on the SDS-PAGE was observed at approximately 30 kDa (Figure 1A). Two-step purification methods using Ni-NTA affinity chromatography followed by gel filtration chromatography yielded protein samples with >95% purity, as determined via visual inspection of the SDS-PAGE gels. Gel filtration chromatogram with the calibration curve of protein sizes (Figure 1B) and SEC-MALS analysis (Figure 1C) suggested a molecular mass of approximately 30.5 kDa, which corresponded to the theoretical mass (30681.5 Da) of the monomer. The far-UV CD spectrum (Figure 1D) was used to determine the extent of secondary structural alterations in BvDOD at diverse pH values. The CD spectrum of the purified recombinant BvDOD protein exhibited well-folded properties and mixed α/β structural characteristics, with approximately 34.3% helix, 16.7% strand, and 11.9% turn conformations predicted using the BeStSel server.^45^ Moreover, within the pH range of 5.5–9.0, BvDOD maintained its secondary and tertiary structure. The CD signal’s intensity change could be attributed to the variations in protein solubility under different pH conditions.

### Enzymatic Activity of BvDOD

Before performing enzyme kinetic measurements, the product of BvDOD with L-DOPA as the substrate was analyzed using HPLC and MS. The HPLC chromatograms (Figure S1A) illustrated three detection modes: the untreated control (red line) was detected at 230 nm for L-DOPA, and the reaction samples were detected at 230 nm (blue line) for L-DOPA and at 424 nm (black line) for betalamic acid. Two peaks were observed in the untreated control, which corresponded to ascorbic acid and the substrate L-DOPA. After the enzymatic reaction with BvDOD, the 230 nm chromatogram (blue line) showed a distinct reduction in the L-DOPA peak, which signified substrate consumption. In addition, the 424 nm chromatogram (black line) revealed a new peak, which could be ascribed to the product betalamic acid. To confirm the identity of this product, the peak corresponding to betalamic acid was collected from HPLC and subjected to MS analysis (Figure S1B). The MS spectrum detected a compound with an *m*/*z* value of 210, which agreed with the molecular weight of betalamic acid.

**Figure 2:**
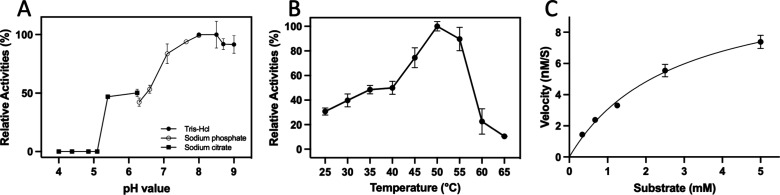
**Effect of various temperatures and pH values on BvDOD** (A) Optimal pH was evaluated by incubating 0.5 μM BvDOD and 2.5 mM L-DOPA in a pH range of 4.0–9.0 for 30 s at 25 °C. (B) Optimal temperature was evaluated by incubating 0.5 μM BvDOD and 2.5 mM L-DOPA at temperatures ranging from 25 to 65 °C for 30 s in pH 8.0. (C) Steady-state kinetics of BvDOD in pH 8.0 at 50 °C was recorded. Kinetic parameters are shown in Table S2.

Enzyme activities of BvDOD on L-DOPA were measured by observing a yellow coloration with a λ_max_ of 424 nm. The optimum pH for the DOPA-dioxygenase activity was established to be 8.5. From pH 8.5 to pH 5.5, the activity of BvDOD decreased gradually by half (Figure 2A). The optimal temperature for BvDOD was 50 °C, and the enzymatic activity decreased rapidly when the temperature exceeded 55 °C (Figure 2B). Subsequently, the dependence of the reaction on substrate concentration was determined to characterize the kinetics of BvDOD. The initial velocity against various L-DOPA concentrations with a fixed concentration of BvDOD was measured in phosphate buffer (pH 8.5) at 50 °C. Kinetic analysis revealed that increased DOPA concentration led to elevated enzymatic activity (Figure S2). A value of 2.734 mM was calculated for the Michaelis–Menten constant K_m_ by fitting the steady-state rates to the Michaelis–Menten equation. In addition, values for the maximum rate and catalytic efficiency were determined as *V*_max_ = 11.42 nM/sec and *k*_cat_ = 0.034260 min^–1^ (Figure 2C and Table S2), respectively.

The Fe K-edge X-ray absorption spectrum of the BvDOD crystal was recorded at beamline TPS05A of the NSRRC, Taiwan (Figure S3). This analysis verified the presence of iron within the crystal and provided insights into its coordination environment. To establish the identity of the metal ion in the active site of BvDOD, ICP-MS was used to analyze the metal content of the purified enzyme. The concentration of the purified enzyme was estimated to be 18.26 nmol per 19.95 nmol of the protein (determined using the Bradford method). The result implied that BvDOD contained one iron. Moreover, the effects of various metal ions (e.g., Fe^3+^, Fe^2+^, Mg^2+^, Ca^2+^, Zn^2+^, Cu^2+^, Ni^2+^, Co^2+^, and Mn^2+^) on BvDOD activity were investigated. The findings showed that BvDOD exhibited the highest catalytic activity in the presence of Fe^2+^, highlighting its role as an essential cofactor for enzymatic function (Figure S4). These combined results demonstrate that iron is the metal ion coordinated in the active site of BvDOD and is imperative for its catalytic activity.

### The Overall Structure of BvDOD

After verifying the functionality of heterologously expressed BvDOD via kinetic analyses, the next objective was to elucidate the protein’s three-dimensional structure, aiming to comprehend its specificity and catalytic mechanism. This effort was significant owing to insufficient structural data for plant L-DOPA-dioxygenase. Crystals were successfully obtained by screening and optimizing the conditions for purified BvDOD (Figure S5) and diffracted to 3.08 Å (Table 1). Despite the absence of a suitable search model for molecular replacement, a partial solution was achieved using the crystal structure of bacterial YgiD (PDB code: 2PW6), which shared only 37% sequence identity with BvDOD. After multiple iterations of model rebuilding, the 2Fo-Fc map exhibited continuous and well-defined electron density of good quality. The crystal structure of BvDOD, which included 263 amino acid residues (Asn6–Thr268) and one Fe atom, was ultimately refined to an R_work_ of 16.70% and an R_free_ of 22.00% (Table 1).

**Table 1:** X-ray Data Collection and Refinement Statistics of BvDOD

	BvDOD (PDB code: 8IN2)
data collection	
wavelength	1.00000
resolution range	26.17–3.08 (3.18–3.08)^a^
space group	P3121
Cell dimensions	
a, b, c (Å)	95.873, 95.873, 124.908
α, β, γ	90, 90, 120
total reflections	86800
unique reflections	12666 (1220)
redundancy	6.8 (6.9)
completeness (%)	99.5 (98.2)
mean I/sigma(I)	26.100 (4.163)
R_merge_^b^	0.078 (0.497)
C*C*_1/2_^c^	97.92(92.00)
Refinement statistics	
R_work_ (%)	16.70(24.22)
R_free_ (%)	22.00(32.45)
macromolecules	4114
ligands	14
protein residues	526
RMS (bonds)	0.011
RMS angles	1.22
Ramachandran plot	
favored (%)	96.17
allowed (%)	3.83
outliers (%)	0
average B-factor	58.70

a Values in parentheses are for highest-resolution shell.

b Rmerge = ΣhΣi|Ih,i-Ih|/ΣhΣiIh,i, where Ih is the mean intensity of the I observations of symmetry related reflections of h.

CC1/2 is the correlation coefficient between intensities from random half-data sets. A derived quantity of CC1/2, is an estimate of the “true” CC of the data under examination to the (unknown) true intensities.

**Figure 3:**
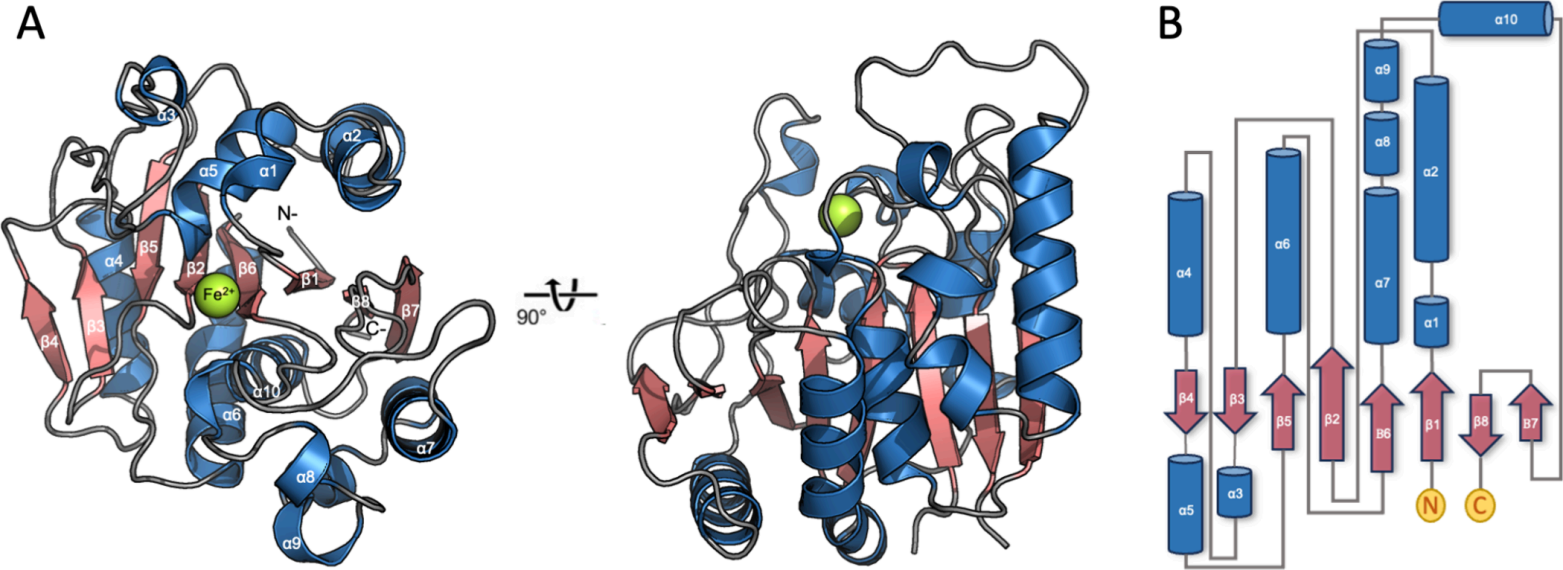
**Overall structure of BvDOD** (A) The overall structure of BvDOD is shown as a cartoon diagram, with secondary structure elements labeled. Helices, strands, and loops are colored blue, red, and gray, respectively. The metal ion is represented as a green sphere. The right-side figure shows a 90° horizontal rotation of the monomer structure from the top-side figure. (B) Topology diagrams use the same color scheme as the cartoon representations.

The enzyme displayed a single-domain structure with a mixed eight-strand β-sheet surrounded by 10 α-helices. The α/β-fold, comprising a central β-sheet flanked by two clusters of α-helices, was a hallmark of its structural architecture (Figure 3A). Secondary structure elements were designated by their sequential order in the primary sequence (Figure 3B). The model’s stereochemistry was robust, with 96.17% of residues in the “most favorable” regions and 3.83% in the “favorable” regions of the Ramachandran diagram, which indicated good model quality (Figure S6). The crystal structure of BvDOD conformed to the *P*3_1_21 space group and accommodated two molecules per asymmetric unit. Superimposition of the Cα coordinates from the two molecules revealed a root-mean-square deviation of 0.186 Å, which highlighted the high degree of structural consistency between the two forms. This slight deviation confirmed minimal structural differences between the crystal structures. In addition, SAXS analysis of BvDOD demonstrated that the protein was present as a monomer in solution (Figure S7). Comprehensive statistics for data collection, phasing, and refinement are summarized in Table 1.

A crucial feature of BvDOD was the presence of an interior pocket containing a mix of hydrophobic and hydrophilic regions accessible from the exterior. This pocket, approximately 20 Å deep and 585 Å^3^ in volume, was strategically positioned to facilitate substrate binding and catalysis. In the substrate-free form, the Fe ion within the active site was coordinated by the oxygen atoms of three water molecules and the Nε2 atoms of three histidine residues (His15, His53, and His229). These residues were strategically located within loops that connected β1-α1, β2-β3, and α9-α10, respectively, playing a crucial role in maintaining the active site’s structural integrity.

### Structure Comparison of BvDOD with Its Relatives

**Figure 4:**
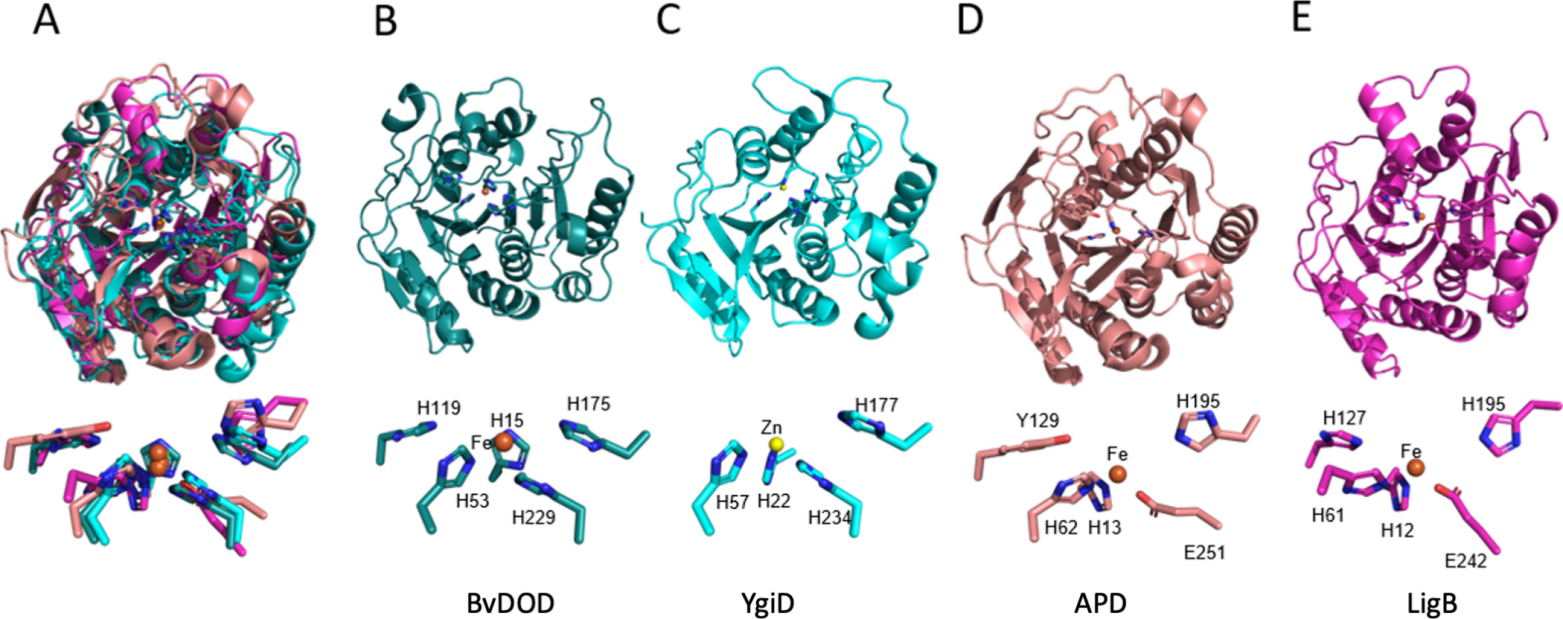
**Structural comparison and metal ion coordination in DOD family members** (A) Superimposition of YgiD (light blue), LigB (light pink), and APD (dark red) on BvDOD (navy blue), revealing significant structural similarity and a common core fold shared by the enzymes. (B-E) Individual structures of BvDOD, YgiD, APD, and LigB, demonstrating conserved architecture and metal iron coordination across these DOD family proteins. Below each structure, a detailed view of metal ion coordination is shown, with iron atoms represented as orange spheres and coordination bonds depicted with light blue dashed lines, demonstrating conserved metal-binding interactions across the superfamily.

To identify structural homologues of BvDOD, a search was conducted using the DALI server^46^ (Table S3). The structural alignments demonstrated that BvDOD shared high structural similarity with several members of the extradiol dioxygenase family, including YgiD (PDB code: 2PW6, Z-score: 29.1%, 32% identity), DHPAO^47^ (PDB code: 8IQ8, Z-score: 25.3%, 18% identity), APD^48^ (PDB code: 3VSH, Z-score: 23.8%, 19% identity), DesB^49^ (PDB code: 3WPM, Z-score: 23.4%, 18% identity), TK2203 (PDB code: 5HEE, Z-score: 23.3%, 14% identity), and LigB^50^ (PDB code: 1B4U, Z-score: 23.3%, 16% identity). These enzymes shared a common structural core, as inferred from the selective superimposition of BvDOD, YgiD, APD, and LigB (Figure 4A), which implied a conserved mechanism within this enzyme family.

The active site coordinated with a divalent metal ion in all these enzymes. Specifically, BvDOD (Figure 4B) and YgiD (Figure 4C) used three conserved histidine residues to coordinate the catalytic metal ion, a characteristic also observed in cysteine dioxygenase and other members of the cupin protein family.^51^ In contrast, APD (Figure 4D) and LigB (Figure 4E) exhibited a different configuration from BvDOD, utilizing two histidine residues and one glutamate for metal ion coordination. Both BvDOD and LigB coordinated a divalent iron ion at their active site. In BvDOD, the coordinating residues were H15, H53, and H229 (Figure 4B, lower panel), whereas in LigB, they were His12, His61, and Glu242 (Figure 4E, lower panel). The iron ion adopted an octahedral coordination geometry, typical of six-coordinate complexes. In addition, two highly conserved residues, His119 and His175, were located near BvDOD’s catalytic center, which corresponded to His127 and His195 in LigB. In LigB, His127, and His195 formed hydrogen bonds with the adjacent hydroxyl groups of the catecholic substrate and stabilized it for catalysis (Figure 4E). Based on these similarities, L-DOPA was hypothesized to bind similarly in BvDOD.

Further structural comparisons indicated distinct substrate specificities among these enzymes. The surface presentation of the BvDOD active site revealed a large, open entrance, in contrast to the smaller, narrower active sites observed in APD and LigB. APD, and LigB were specialized for smaller substrates, such as 2-aminophenol and protocatechuate, respectively (Figure S8). These structural differences suggested that while BvDOD can accommodate larger catecholic compounds such as L-DOPA, LigB, and APD are adapted for processing smaller substrates.

### Structure-Guided Site-Directed Mutagenesis

**Figure 5:**
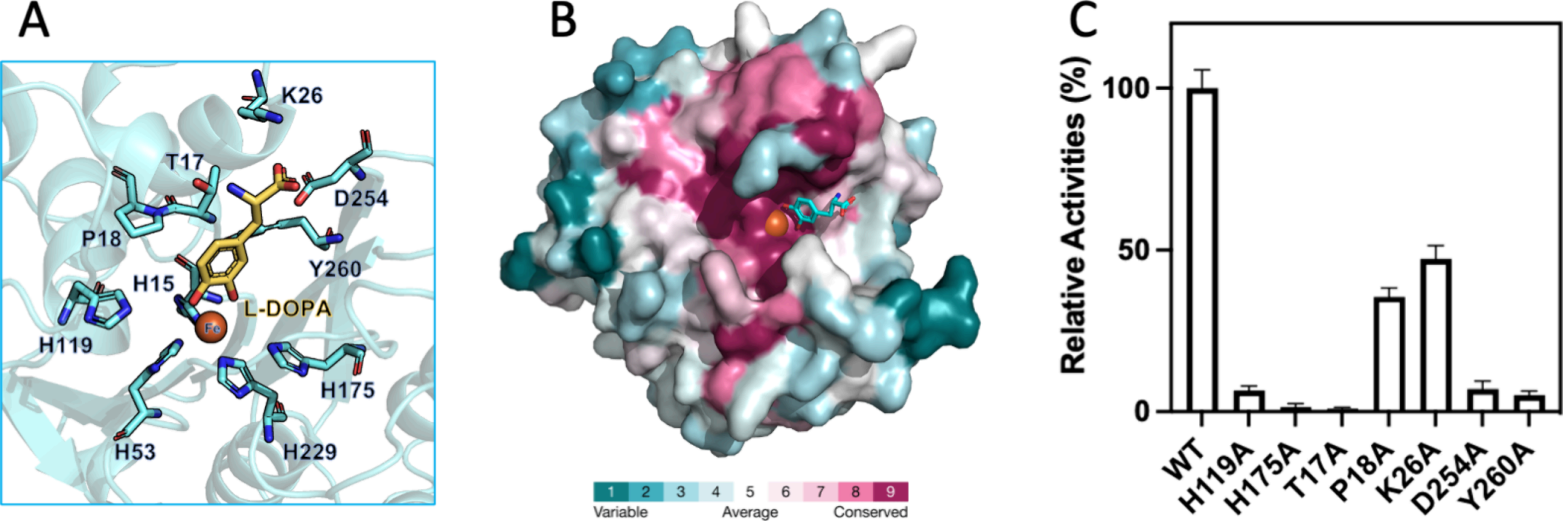
**Structure-guided mutagenesis for functional analysis of BvDOD** (A) The molecular docking model shows L-DOPA (yellow) bound to BvDOD, with key residues in the binding pocket or interacting with the metal ion highlighted in cyan. The iron atom is depicted as an orange sphere. (B) ConSurf analysis of BvDOD-DOPA shows that the residues involved in recognizing the amino acid portion of L-DOPA are not conserved in plant DOD enzymes. (C) Site-directed mutants of BvDOD, based on the structural model, were created and their enzymatic activities were tested.

In silico docking experiments were conducted using AutoDock Vina to elucidate the interaction between BvDOD and its substrate L-DOPA. The resulting BvDOD–L-DOPA complex with the minimum binding energy was proposed (Figure 5A). Docking pose analysis revealed that L-DOPA’s O3 and O4 atoms coordinated with Fe ion and formed hydrogen bonds with His175 and His119, respectively.

BvDOD–L-DOPA complex analysis using the ConSurf server^52^ indicated that amino acid residues within the L-DOPA binding pocket were not conserved. This observation suggested that DOD family members may utilize diverse substrates driven by variability in the residues forming the binding pocket (Figure 5B). In other words, the lack of conservation at the binding site suggested potential functional diversity among DOD enzymes, allowing them to accommodate diverse substrates or perform distinct catalytic roles. In contrast, two highly conserved residues, His175 and His119, were positioned near the two hydroxyl groups of L-DOPA, highlighting their crucial role in stabilizing or catalyzing ligands with catechol rings as the common feature. Site-directed mutagenesis was performed to investigate their specific contributions, replacing these residues with alanine. As expected, the H175A and H119A mutants exhibited a considerable loss of catalytic activity, which confirmed the essential role of His175 and His119 in enzymatic function (Figure 5C). This dramatic activity reduction emphasized their importance in maintaining the active site’s structural integrity and facilitating substrate interactions.

Five additional binding residues were mutated to alanine to investigate L-DOPA specificity to assess their impact on enzyme activity. The docking model identified Thr17, Pro18, Lys26, Asp254, and Tyr260 around the L-DOPA binding pocket (Figure 5A). These residues were mutated, and their enzymatic activities were examined. The T17A, D254A, and Y260A mutants showed a near-complete activity loss compared with the wild-type protein (Figure 5C), implying that Thr17, Asp254, and Tyr260 were crucial for catalytic efficiency. This activity loss suggested that these residues played key roles in substrate orientation or stabilization within the active site. Conversely, the K26A mutant retained approximately 50% of the wild-type activity (Figure 5C), indicating that while Lys26 was important, it was not as crucial for catalytic function as the other residues studied. This partial retention of activity signified that Lys26 may play a supportive or secondary role in substrate binding or catalysis. Moreover, the P18A mutant retained only 40% activity (Figure 5C), possibly due to the perturbation of Thr17’s orientation, supporting the role of Thr17 in substrate recognition.

To further understand the impact of these mutations on *k*_cat_ or *K*_m_, the enzyme kinetics of the mutants were analyzed (Table S2). As expected, only the P18A and K26A mutants permitted the measurement of kinetic parameters. Interestingly, compared with wild-type BvDOD, the P18A and K26A mutants exhibited similar affinity for L-DOPA but significantly reduced catalytic efficiencies of 14.59 and 44.75 min^–1^, respectively. Furthermore, despite their relatively low activities, attempts were made to measure the kinetic parameters of the D254A and Y260A mutants. D254A and Y260A exhibited dramatically reduced catalytic efficiencies of 5.99 and 4.11 min^–1^, respectively.

Detailed kinetic analysis (Table S2) indicated the pivotal roles of Thr17, His119, His175, Asp254, and Tyr260 in the catalytic mechanism of BvDOD and the relative resilience of the enzyme’s function to mutations at Pro18 and Lys26. These mutagenesis experiments provided valuable insights into the functional architecture of BvDOD and underscored the importance of specific amino acid residues in enzyme activity. The dramatic loss of function in the T17A, H119A, H175A, D254A, and Y260A mutants highlighted the delicate balance required for effective catalysis and the precise positioning of key residues within the active site.

### Proposed Mechanism for Extradiol Ring Cleavage of L-DOPA by BvDOD

Understanding the structural details of BvDOD offered beneficial insights into its catalytic mechanism. The Fe ion at the active site was essential for the enzyme’s dioxygenase activity and facilitated the incorporation of molecular oxygen into the substrate. The coordination environment of the Fe ion, with histidine residues and water molecules, suggested a mechanism in which substrate binding displaced the water molecules, positioning the catecholic substrate for oxidative cleavage. The hydrophobic/hydrophilic pocket further indicated a selective substrate binding process, which contributed to the enzyme’s specificity.

**Figure 6:**
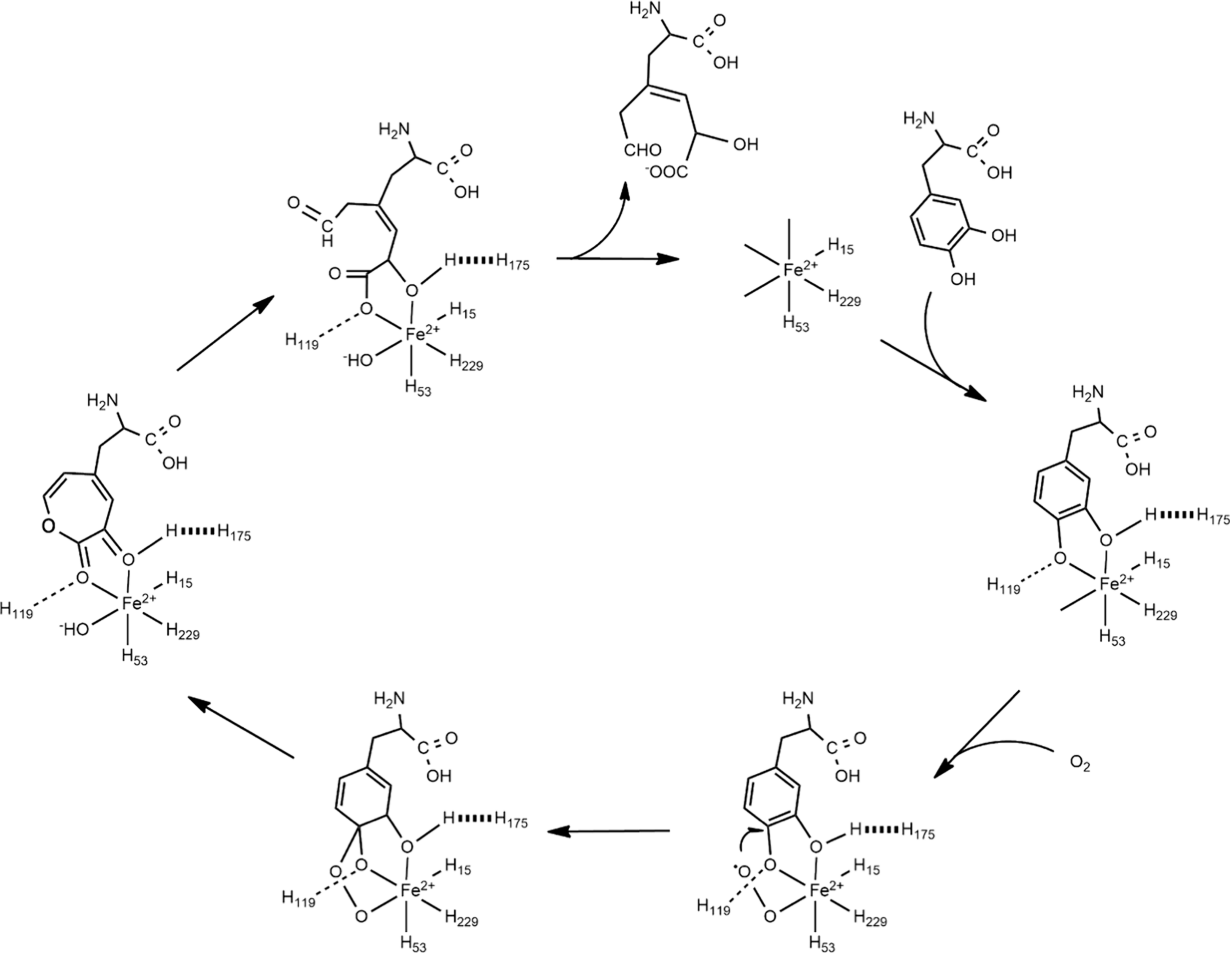
General mechanism for extradiol ring cleavage of L-DOPA by BvDOD.

A general catalytic mechanism was proposed. In the initial catalytic step of this mechanism (Figure 6), the residues His15, His53, and His229 of BvDOD coordinated with the ferrous ion (Fe^2+^). Subsequently, the catecholic substrate bound to the ferrous ion within the active site. The His119 residue of the enzyme catalyzed the deprotonation of L-DOPA and mediated its coordination to the ferrous ion, which promoted the binding of O_2_ to the Fe ion.

The formation of an Fe^2+^-bound oxygen species resulted in the transfer of an electron from Fe^2+^ to O_2_, yielding an Fe^3+^-superoxide species. This superoxide species enabled the subsequent transfer of an electron from L-DOPA to the Fe^3+^ center, forming an alkylperoxo intermediate. This intermediate underwent O–O bond cleavage, which led to the formation of an unsaturated lactone intermediate via a Criegee rearrangement.^53−55^ The lactone ring was then hydrolyzed, yielding 4,5-seco-DOPA, which spontaneously cyclized to form the reaction product betalamic acid.

## Discussion

BvDOD and LigB belong to the same superfamily of class III extradiol dioxygenases. The enzyme LigB catalyzes the ring-opening reaction of 3,4-dihydroxybenzoic acid. His127 forms hydrogen bonds with the substrate and stabilizes and positions it for the reaction. His195 acts as a Lewis acid and facilitates the reaction. Similarly, in BvDOD, His119 is speculated to form a hydrogen bond with the oxygen atom on the fourth carbon of L-DOPA, which stabilizes the substrate and positions it appropriately for catalysis. Mutation of His119 to alanine (H119A) leads to a loss of enzymatic activity, which indicates its crucial role. In addition, His119 is proposed to deprotonate the hydroxyl group on the fourth carbon of L-DOPA, which makes the negatively charged L-DOPA more likely to bind to the ferrous ion (Fe^2+^). Furthermore, His175 acts as a Lewis acid and loses activity when mutated to alanine (H175A), which emphasizes its role in the catalytic process. Five residues (Thr17, Pro18, Lys26, Asp254, and Tyr260) around the binding pocket likely influence the enzyme activity. The mutations possibly affect the orientation or stabilization of the substrate in the active site without considerably impacting its binding affinity, resulting in a similar *K*_m_ but reduced *k*_cat_. If the substrate interacts normally with the iron center via chelation, improper positioning or weakened coordination with iron owing to these mutations could result in inefficient oxygen activation or substrate transformation, decreasing the overall catalytic turnover.

YgiD, a DOD homologue from *E. coli*, also catalyzes the conversion of L-DOPA. Its crystal structure has been established, showing its ability to convert L-DOPA into 2,3-seco-DOPA, which cyclizes spontaneously to form muscaflavin.^29^ YgiD and BvDOD proteins were compared, which revealed that although their amino acid sequences differ, their folded structures are remarkably similar. The active site pocket of YgiD is larger than that of BvDOD. BvDOD contains a residue that might hinder the catalytic reaction of 2,3-seco-DOPA; however, this finding requires further experimental validation.

In this study, BvDOD was expressed in *E. coli*, purified to homogeneity, and characterized for specific biochemical properties, including enzyme kinetics. BvDOD belongs to the extradiol dioxygenase superfamily and catalyzes the conversion of L-DOPA into betalamic acid. This enzyme demonstrates optimal activity at pH 8.0, with kinetic values of *K*_m_ = 2.734 mM and *V*_max_ = 11.42 nM/s. The *K*_m_ value is lower than those of 4,5-DOPA dioxygenases from other species, for example, 4.5 mM for DODA from *Amanita muscaria*(28) and 7.9 mM for YgiD from *E. coli,*(29) which implies a higher substrate affinity. This is the first report on the structure of a plant DOD from the best-known betalain-producing *B. vulgaris*, revealing the molecular mechanisms of substrate binding. These findings provide valuable structural information for future application in betalain synthesis.

## Supporting Information Available

The Supporting Information is available free of charge on the ACS Publications Web site. Table S1. Primers used in this study. Table S2. Kinetic parameters of BvDOD and mutants. Table S3. Structural comparison of BvDOD with structural relatives. Figure S1. Analysis of BvDOD enzymatic reaction using HPLC and MS. Figure S2. Kinetic analysis of BvDOD. Figure. S3. Iron K-edge X-ray absorption spectrum of BvDOD. Figure S4. Effects of some metal ions on BvDOD activity. Figure S5. Crystals of BvDOD. Figure S6. Ramachandran plot analysis. Figure S7. Solution SAXS analysis of BvDOD. Figure S8. Surface presentation of dioxygenase enzymes (PDF)

## Accession Codes

Final coordinates and structure factors for BvDOD have been deposited in the protein data bank (PDB) with the accession code 8IN2.

## Abbreviations

DOPA: dihydroxyphenylalanine
BvDOD: 4,5-DOPA-extradiol-dioxygenase from *Beta vulgaris*
Ni-NTA: nickel nitrilotriacetic acid
IPTG: isopropyl β-D-1-thiogalactopyranoside
SDS-PAGE: sodium dodecyl sulfate-polyacrylamide gel electrophoresis
PCR: polymerase chain reaction
FPLC: fast protein liquid chromatography
CD: circular dichroism
SEC-MALS: size-exclusion chromatography-multiangle light scattering
SAXS: small-angle X-ray scattering
WT: wild-type.
