# Supplementary material for: Structural Insights into 4,5-DOPA Extradiol Dioxygenase from Beta vulgaris: Unraveling the Key Step in Versatile Betalain Biosynthesis
Source: J Agric Food Chem. 2025 Mar 7;73(11):6785–94. doi: 10.1021/acs.jafc.4c09501 (PMC11926856; doi:10.1021/acs.jafc.4c09501)
Supplement: Supplementary file 1 — jf4c09501_si_001.pdf [file jf4c09501_si_001.pdf]

Supporting Information

Structural Insights into 4,5-DOPA Extradiol Dioxygenase from Beta vulgaris: Unraveling the Key Step in Versatile Betalain Biosynthesis

Chih-Chia Chiang[a,1], Yen-Ju Lu[a,b,1], Jia-Wei Liu[a], Sheng-Wei Lin[b], Chun-Chi Chou[a], Chia-Hsin Lin[b], I-Weh Chien[a], and Chun-Hua Hsu[a,b,c,d*]

[a]Department of Agricultural Chemistry, National Taiwan University, Taipei 10617, Taiwan

[b]Institute of Biochemical Sciences, National Taiwan University, Taipei 10617, Taiwan

[c]Genome and Systems Biology Degree Program, National Taiwan University and Academia Sinica, Taipei 10617, Taiwan

[d]Center for Computational and Systems Biology, National Taiwan University, Taipei 10617, Taiwan

[1]These authors contributed equally to this work.

*Corresponding authors

Chun-Hsu Hsu

Email: andyhsu@ntu.edu.tw; Tel: +886-2-33664468

Table S1. Primers used in this study

**mutants**	**primer (F)**	**primer (R)**
T17A	5’-GCGCCCCGATGATGGCAATTGACGAC-3’	5’-CGTGGCTGATAAAAAAGGTTTCTTTGATG-3’
P18A	5’-GCACCGCCATGATGGCAATTGACGAC-3’	5’-CGTGGCTGATAAAAAAGGTTTCTTTGATG-3’
K26A	5’-GACGACAGCGCCCCGAGCAAG-3’	5’-AATTGCCATCATCGGGGTGC-3’
H119A	5’-GTCGTGGGTTCGACGCCAGCTCATGGGTGC-3’	5’-GCACCCATGAGCTGGCGTCGAACCCACGAC-3’
H175A	5’-TCTGGGGGTGCAGTGGCCCCTTCTGATGACAC-3’	5’-GTGTCATCAGAAGGGGCCACTGCACCCCCAGA-3’
D254A	5’-CTGATCCACAATAGCTGGGCAGGCGGTATCATG-3’	5’-CTCGGCCTTGCTATTTTCACCTGCGG-3’
Y260A	5’-CATGAGCGCCGGCAGCTATAAATTTAC-3’	5’-ATACCGCCATCCCAGCTATTG-3’

Table S2. Kinetic parameters of BvDOD and mutants

enzymes	V_{max} (nM sec^{-1})	*Km* (mM)	k_{cat} (min^{-1})	k_{cat}/Km (min^{-1} mM^{-1})
WT	11.42	2.734	0.034260	0.012530
H119A	N.D.	N.D.	N.D.	N.D.
H175A	N.D.	N.D.	N.D.	N.D.
T17A	N.D.	N.D.	N.D.	N.D.
P18A	2.432	2.368	0.007295	0.003082
K26A	7.459	2.886	0.022375	0.007752
D254A	0.9988	3.108	0.002995	0.000964
Y260A	0.6850	1.812	0.002055	0.001134

N.D.: not determined

Table S3. Structural comparison of BvDOD with structural relatives using DALI server

PDB code	Protein	Ca atoms	Z-score	RMSD	Identity (%)
2PW6	ygiD<br>Uncharacterized protein<br>(E. coli)	234	29.1	2.0	32
8IQ8	DHPAO<br>3,4-dihydroxyphenylacetate 2,3-dioxygenase<br>(Acinetobacter baumannii)	252	25.3	2.8	18
3VSH	APD<br>2-Animophenol-1,6-Dioxygenase<br>(Comamonas testosteroni CNB-1)	250	23.8	2.9	19
3WPM	DesB<br>Extradiol Dioxygenase<br>(Sphingobium sp. SYK-6)	239	23.4	2.6	18
5HEE	TK2203 protein<br>Putative extradiol dioxygenase<br>(Thermococcus kodakarensis KOD1)	239	23.3	2.9	14
1B4U	LigAB<br>protocatechuate 4,5-dioxygenase<br>(Sphingomonas paucimobilis)	238	23.3	2.6	16
3BCZ	Memo<br>Peptide binding protein<br>(Homo sapiens)	215	16.2	3.2	13

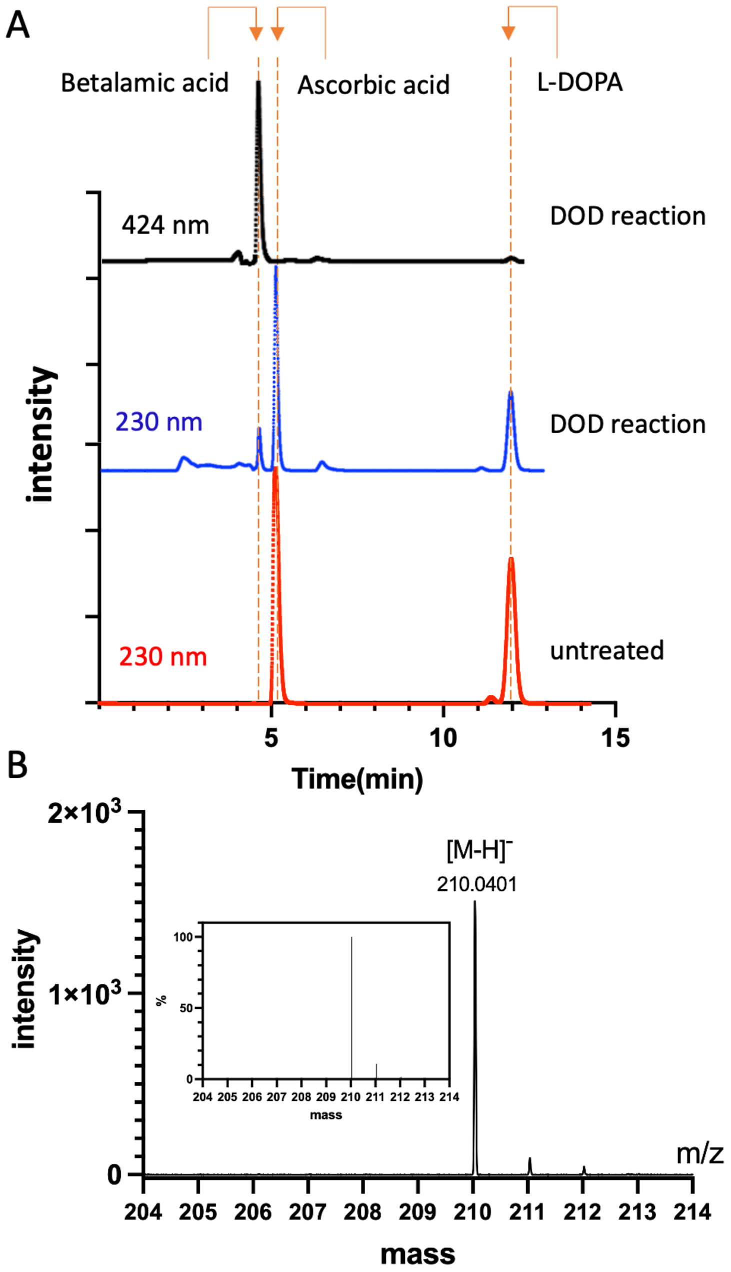

Figure S1. Analysis of BvDOD enzymatic reaction by HPLC and MS. (A) HPLC chromatograms of the untreated control (red, detected at 230 nm), the DOD reaction product detected at 230 nm (blue), and at 424 nm (black). Peaks corresponding to ascorbic acid, L-DOPA, and betalamic acid are labeled. The formation of betalamic acid is confirmed by the appearance of a new peak at 424 nm following the DOD reaction. (B) Mass spectrum of the collected product from the 424 nm peak, identifying betalamic acid with an m/z value of 210. The inset shows the theoretical mass spectrum of betalamic acid.

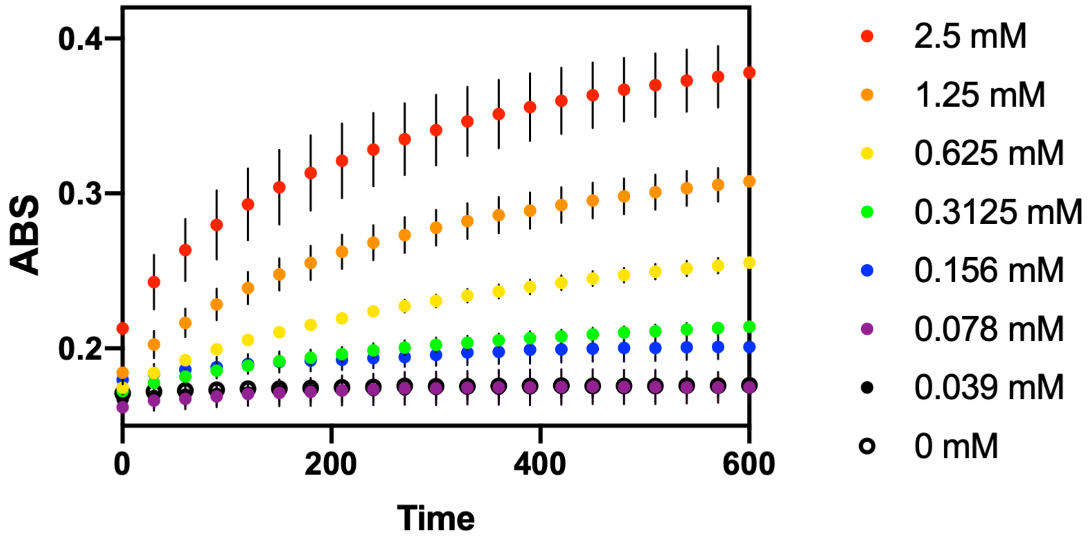

Figure S2. Kinetic analysis of BvDOD indicates that increasing concentrations of DOPA result in a corresponding increase in enzymatic activity, as shown in the graph. The data illustrate a clear relationship between substrate concentration and enzyme velocity, supporting the model of classic enzyme-substrate kinetics.

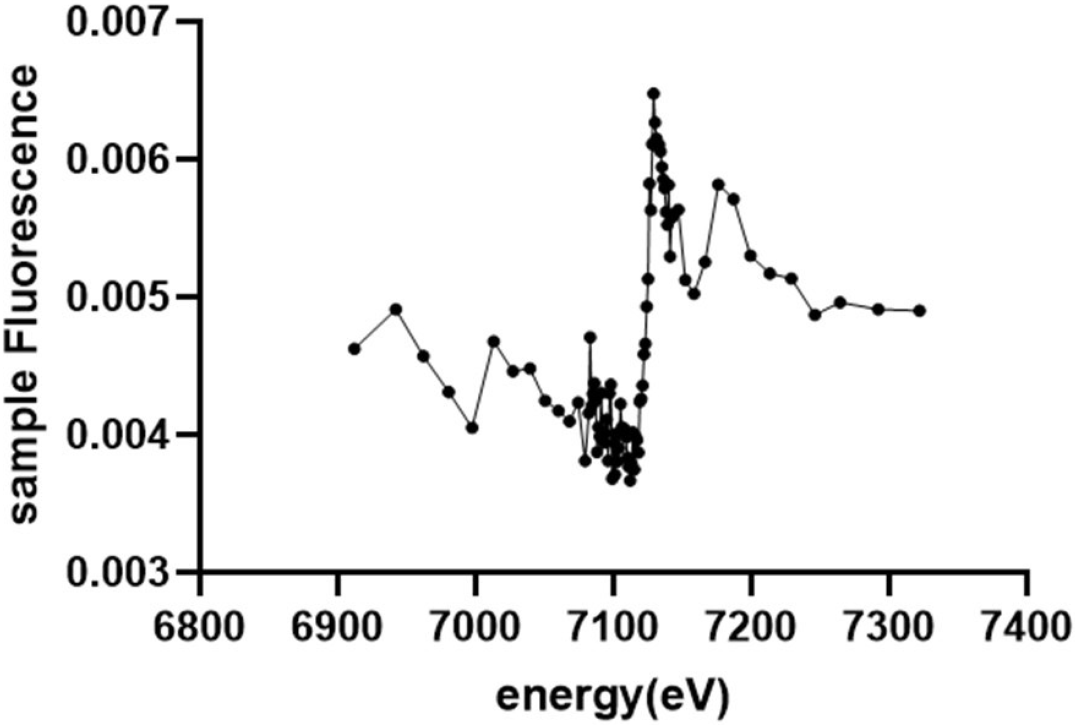

Figure S3. Iron K-edge x-ray absorption spectrum of BvDOD.

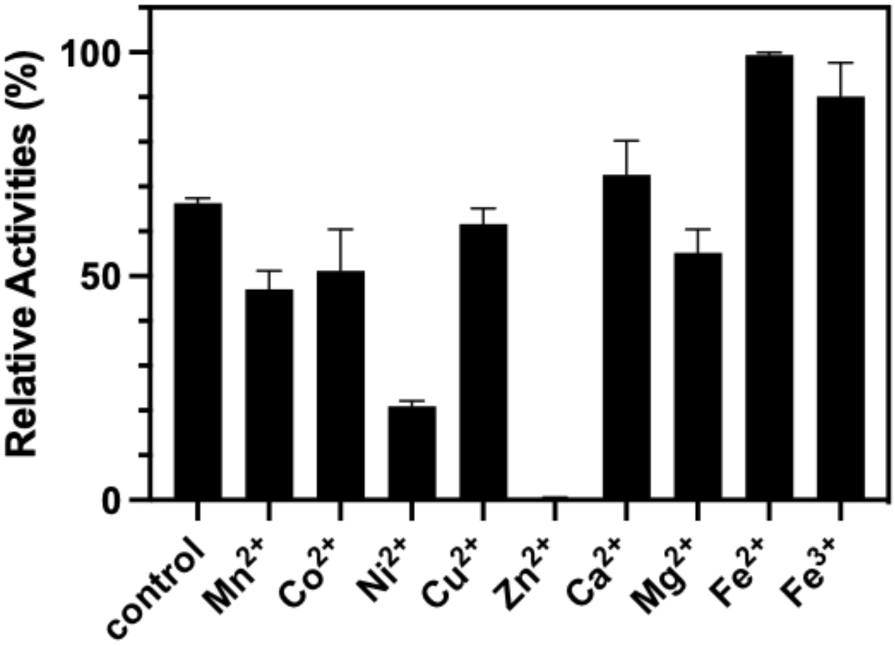

Figure S4. Effects of some metal ions on BvDOD activity.

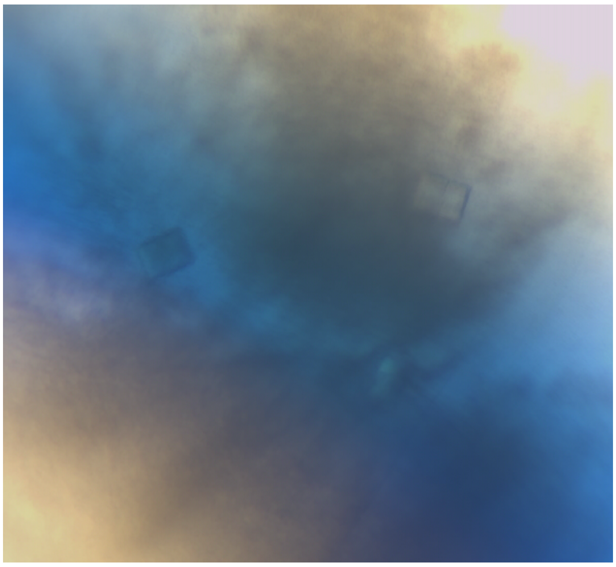

Figure S5. Crystals of BvDOD were grown on protein amorphous precipitation.

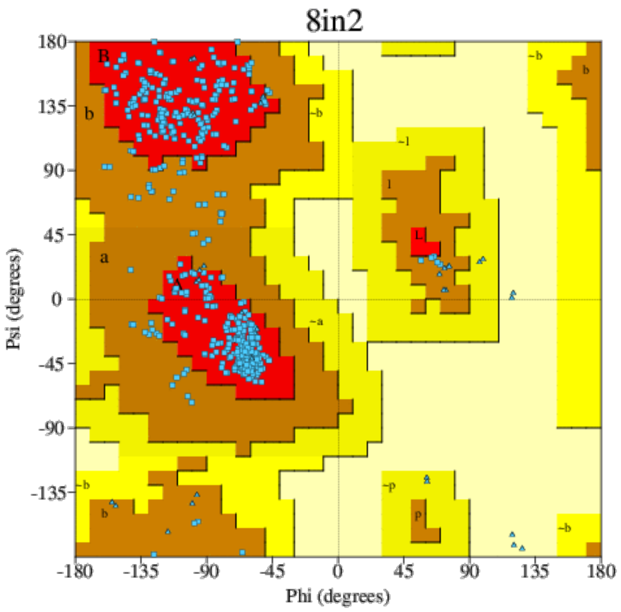

Figure S6. Ramachandran plot analysis showed the good geometry quality of the BvDOD crystal structure.

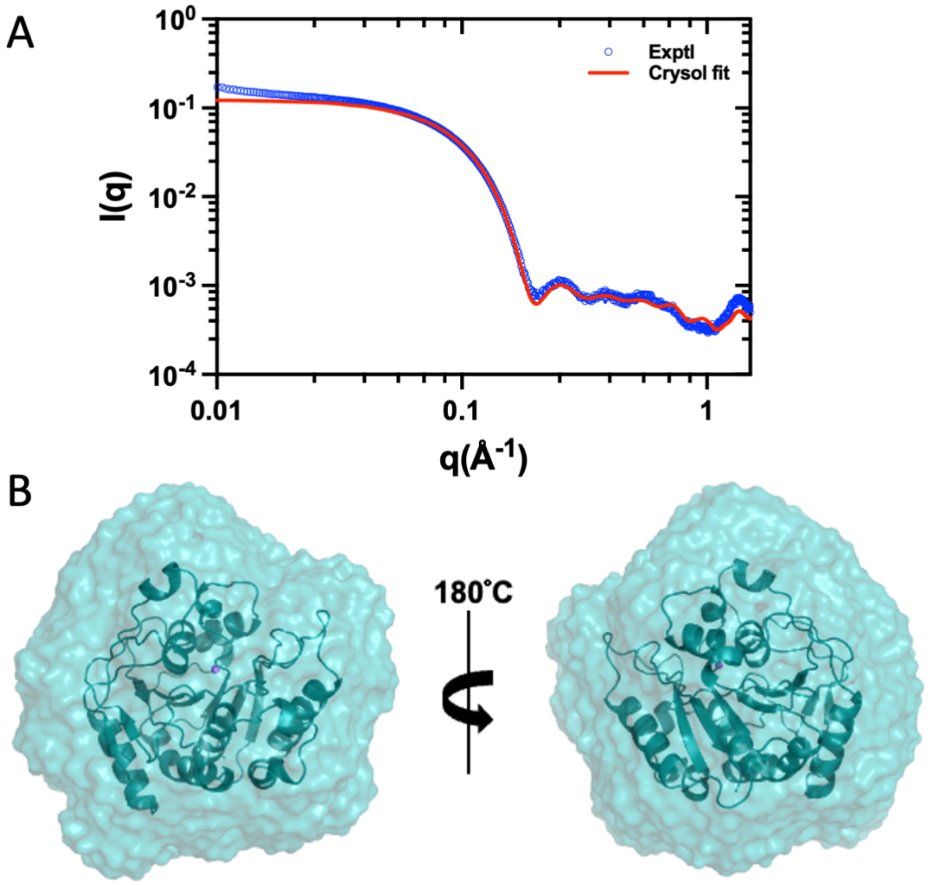

Figure S7. Solution SAXS analysis of BvDOD. (A) The processed SAXS data (blue dot) were fitted with the software ATSAS CRYSOL (red line), and were exported the relationship between q and $I(q)$ profile. (B) Comparison of the structure of BvDOD in solution and the crystal structure was analyzed using WAXSIS and shown as partially transparent surface model; crystal structure is shown as cartoon model.

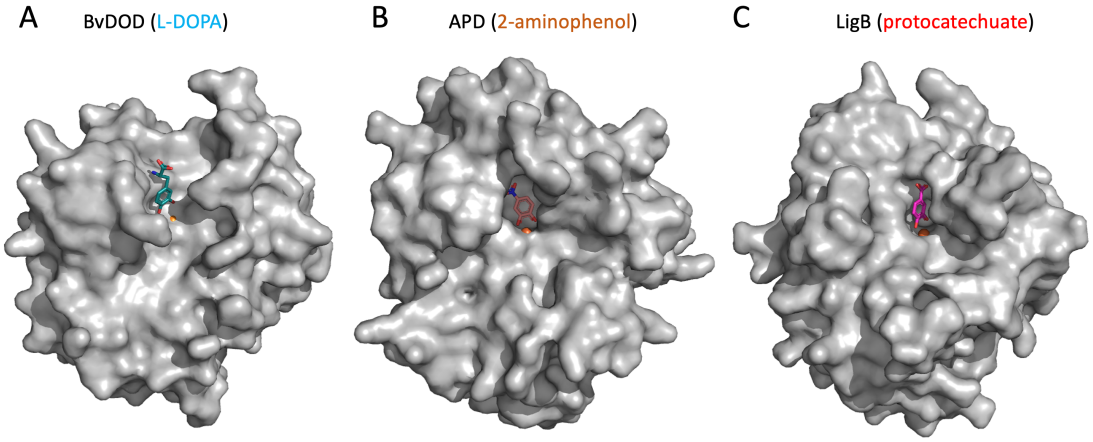

Figure S8. Surface presentation of dioxygenase enzymes. (A) BvDOD displays a widely open entrance, with the ligand L-DOPA shown as cyan sticks. (B) APD features a narrow, compact pocket for substrate accommodation, with the ligand 2-aminophenol represented as orange sticks. (C) LigB also exhibits a narrow, confined pocket for substrate binding, with the ligand protocatechuate depicted as pink sticks.
